# Hepatic Sinusoidal Obstruction Syndrome Induced by Pyrrolizidine Alkaloids from *Gynura segetum*: Mechanisms and Therapeutic Advances

**DOI:** 10.3390/molecules31030410

**Published:** 2026-01-25

**Authors:** Zheng Zhou, Dongfan Yang, Tong Chu, Dayuan Zheng, Kuanyun Zhang, Shaokui Liang, Lu Yang, Yanchao Yang, Wenzhe Ma

**Affiliations:** 1 State Key Laboratory of Mechanism and Quality of Chinese Medicine, Faculty of Chinese Medicine, Macau University of Science and Technology, Macau SAR 999078, China; 3250001617@student.must.edu.mo (Z.Z.); 3230006131@student.must.edu.mo (D.Y.); 3230007737@student.must.edu.mo (T.C.); 3230006143@student.must.edu.mo (D.Z.); 3240000989@student.must.edu.mo (K.Z.); 3240008843@student.must.edu.mo (S.L.); 3240008140@student.must.edu.mo (L.Y.); 2220023685@student.must.edu.mo (Y.Y.); 2 Zhuhai MUST Science and Technology Research Institute, Macau University of Science and Technology, Hengqin Guangdong-Macao In-Depth Cooperation Zone, Zhuhai 519099, China

**Keywords:** *Gynura segetum*, pyrrolizidine alkaloids, hepatic sinusoidal obstruction syndrome, mechanism, therapeutic treatment, RUCAM

## Abstract

The traditional Chinese medicinal herb *Gynura segetum* is increasingly recognized for its hepatotoxic potential, primarily attributed to its pyrrolizidine alkaloid (PA) content. PAs are a leading cause of herb-induced liver injury (HILI) in China and are strongly linked to hepatic sinusoidal obstruction syndrome (HSOS). This review systematically summarizes the pathogenesis, diagnostic advancements, and therapeutic strategies for PA-induced HSOS. Molecular mechanisms of PA metabolism are detailed, encompassing cytochrome P450-mediated bioactivation and the subsequent formation of pyrrole-protein adducts, which trigger sinusoidal endothelial cell injury and hepatocyte apoptosis. Advances in diagnostic criteria, including the Nanjing Criteria and the Roussel Uclaf Causality Assessment Method (RUCAM)-integrated Drum Tower Severity Scoring System, are discussed. Furthermore, emerging biomarkers, such as circulating microRNAs and pyrrole-protein adducts, are examined. Imaging modalities, such as contrast-enhanced computed tomography (CT) and gadolinium ethoxybenzyl diethylenetriamine pentaacetic acid (Gd-EOB-DTPA) magnetic resonance imaging (MRI), have evolved from descriptive tools into quantitative and prognostic instruments. Therapeutic approaches have evolved from supportive care to precision interventions, including anticoagulation, transjugular intrahepatic portosystemic shunt (TIPS), and autophagy-modulating agents. A comprehensive literature review, utilizing databases such as PubMed and Web of Science, was conducted to summarize progress since the introduction of the “Nanjing Guidelines”. Ultimately, this review underscores the critical need for integrated diagnostic and therapeutic frameworks, alongside enhanced public awareness and regulatory oversight, to effectively mitigate PA-related liver injury.

## 1. Introduction

*Gynura segetum* (Lour.) Merr. (GS), a perennial herb of the Asteraceae family, is widely recognized in China by its common names “Tu-San-Qi” or “Ju-San-Qi”. Its medicinal use in China dates back to the Ming Dynasty, where its traditional therapeutic properties were reported to include promoting blood circulation, achieving hemostasis, detoxification, and alleviating edema [1,2]. However, reports of *G. segetum*-associated hepatotoxicity have been prevalent. This toxicity is primarily attributed to its pyrrolizidine alkaloid (PA) content, as established in various studies, placing PAs among the most frequent causes of herb-induced liver injury (HILI) in China [3]. Critically, studies have indicated that a single ingestion of as little as 10 g of GS can induce liver injury, which may rapidly progress to severe hepatic sinusoidal obstruction syndrome (HSOS), also known as veno-occlusive disease (VOD) [4,5].

In Western countries, HSOS is most commonly reported as a complication of hematopoietic stem cell transplantation (HSCT), with an incidence typically ranging from 5% to 20% and a mortality rate exceeding 80% in severe cases. The principal pathogenic mechanism, as detailed in previous studies [6], involves sinusoidal endothelial detachment and swelling induced by radiotherapy or chemotherapy, leading to sinusoidal blockage. In contrast, ingestion of PA-containing herbal remedies constitutes the predominant etiology in China and several other Asian regions. Studies have consistently documented *G. segetum* and its closely related “Tu-San-Qi” species as major causative agents for HSOS, primarily owing to their high PA content. As reported by Lin, G et al., the defining histopathological feature is selective injury to hepatic sinusoidal endothelial cells, which results in non-thrombotic occlusion of sinusoids and subsequent post-sinusoidal portal hypertension [4]. Clinically, the disease typically manifests with acute right upper quadrant pain, hepatomegaly, ascites, and rapid weight gain resulting from fluid retention. To date, no specific antidote for PA-induced HSOS has been identified. Progression to hepatic failure is associated with a markedly elevated mortality rate [7], underscoring the critical importance of early recognition, accurate diagnosis, and immediate cessation of toxin exposure.

Diagnosing PA-HSOS remains challenging. This diagnostic complexity arises because many patients lack a definitive history of exposure to PA-containing substances. Furthermore, the clinical presentation of PA-induced HSOS is known to overlap with other conditions that manifest sinusoidal portal hypertension, such as Budd-Chiari syndrome and right heart failure, which operate through distinct pathophysiological mechanisms. Currently, no effective pharmacological therapy has been established to reverse the hepatotoxicity of *G. segetum* or restore hepatic parenchymal integrity. The diagnosis of drug-induced liver injury (DILI) and herb-induced liver injury (HILI) is further hampered by the absence of reliable biomarkers for routine clinical application [8]. Alarmingly, a recent case report highlighted a four-year-old child who developed PA-HSOS after accidental ingestion of GS [9], underscoring the urgent need to raise public awareness of its potential toxicity. While comprehensive reviews have addressed the general mechanisms and global aspects of 1,2-unsaturated PA-induced HSOS [10], the majority of cases in China and Asia are specifically attributed to *Gynura segetum* (“Tu-San-Qi”). This specific etiology presents unique challenges in pathogenesis, diagnosis, and management. However, recent advances since 2021—including the Drum Tower Severity Scoring System, quantitative imaging biomarkers, and targeted therapies—have not been systematically reviewed within this specific context. This review aims to fill this critical gap by providing a focused update on *G. segetum*-induced PA-HSOS. It emphasizes molecular mechanisms, diagnostic refinements, and therapeutic strategies to guide clinical practice and improve patient outcomes.

*Gynura segetum* (GS) possesses a long therapeutic history as a medicinal plant but poses a significant risk of severe hepatotoxicity. Hepatic sinusoidal obstruction syndrome (HSOS), induced by its pyrrolizidine alkaloid (PA) components, represents a complex pathological process spanning botany, toxicology, and clinical medicine. Drawing upon a systematic literature search of scholarly databases (e.g., PubMed and Web of Science), this review synthesizes advancements and insights related to PA-HSOS, particularly those reported since the inception of the “Nanjing Guidelines”. Specifically, it summarizes the molecular mechanisms underlying PA-induced hepatotoxicity, current diagnostic strategies (encompassing conventional criteria and emerging biomarkers), and potential therapeutic interventions. Ultimately, this review aims to provide insights that enhance the understanding and management of this clinically challenging disease.

## 2. Food and Pharmaceutical Safety Recommendations Regarding PAs

The first documented case of PA-induced HSOS occurred in 1920, linked to the consumption of PA-contaminated wheat [11]. Since then, numerous reports have documented PA-related safety issues globally, encompassing countries such as Afghanistan, the United Kingdom, China, Germany, Hong Kong, India, Jamaica, South Africa, Switzerland, and the United States, with an estimated 8160 poisoning cases recorded to date. This substantial public health burden has subsequently prompted many countries to establish regulations restricting or prohibiting the use of PA-containing herbs [12,13]. The International Agency for Research on Cancer (IARC) has classified PAs as carcinogenic substances. Existing literature indicates that over 660 PAs have been identified across more than 6000 plant species. These compounds function as naturally occurring phytotoxins, serving as chemical defenses against herbivores, and are prevalent in thousands of species from families including Asteraceae, Boraginaceae, and Fabaceae. Many PA-containing plants are either utilized medicinally or serve as contaminants in food products such as honey, tea, and cereals, thus posing a significant public health concern [14,15]. PAs naturally coexist with their more water-soluble N-oxides (PAN-oxides). The ratio between these forms is known to fluctuate within a single plant species, influenced by environmental conditions and growth stage [16,17]. In response to these concerns, the European Food Safety Authority (EFSA) has implemented systematic monitoring and established regulatory limits for PAs and PAN-oxides in contaminated foods to maintain minimal concentrations throughout the food chain. EFSA also established a reference point of 237 µg/kg body weight per day to assess the carcinogenic risk associated with PA exposure. Their assessment concluded that PAs may pose health concerns for regular consumers of tea and herbal infusions, particularly among younger populations. Similarly, the UK Medicines and Healthcare Products Regulatory Agency (MHRA) proposed the inclusion of Senecio-containing products on the list of prohibited substances. This regulatory action makes the sale, supply, or import of unlicensed oral preparations containing such plants illegal within the UK [18]. However, it is recognized that not all PAs exhibit toxicity, with their harmful potential largely dependent on specific structural features of the molecule. Consequently, a comprehensive international regulatory framework to effectively prevent PA exposure and its associated hepatotoxicity remains to be established.

## 3. Metabolism of PAs

**Figure 1:**
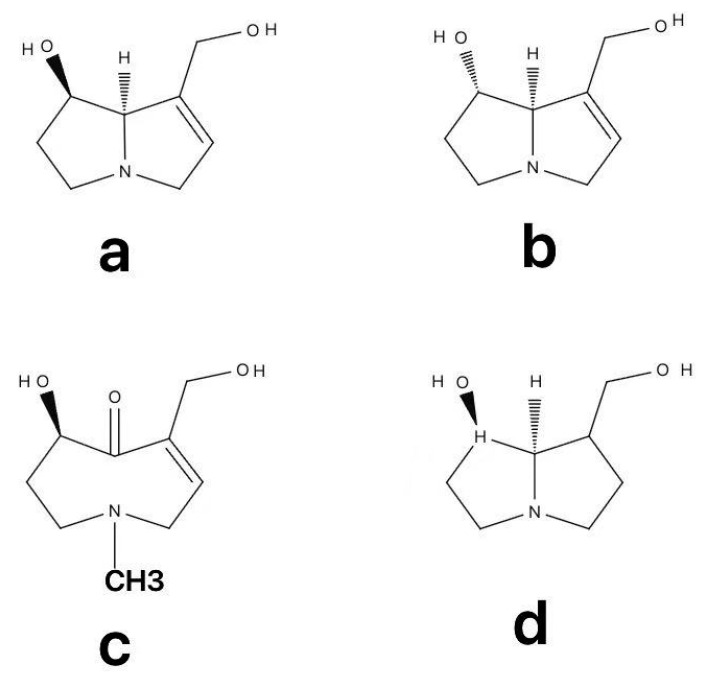
Structures of the most common necine bases. (**a**) retronecine, (**b**) heliotridine, (**c**) otonecine, and (**d**) platynecine.

Pyrrolizidine alkaloids (PAs) are esters comprising a necine base—an amino alcohol characterized by two fused five-membered rings with a shared nitrogen atom—and one or more necic acid moieties. These compounds are known to be toxic to humans and other mammals. PAs are broadly classified into four structural forms based on their N-oxidation state and pyrrolizidine ring saturation: three tertiary amines (saturated, unsaturated, and otonecine types) and one N-oxide form. Key examples include (a) retronecine, (b) heliotridine, (c) otonecine, and (d) platynecine (Figure 1). Structurally, PA molecules typically feature two fused five-membered rings with a shared nitrogen atom at the C-4 position. Most naturally occurring PAs are derivatives of 1-methylpyrrolizidine; however, some exist as 1-hydroxymethyl-1,2-dehydropyrrolizidine esters, which are known to exhibit hepatotoxic properties. The core structure understood to be responsible for PA-induced hepatotoxicity is characterized by an unsaturated necine base containing a 1,2-double bond, one or two hydroxyl groups, and esterified side chains derived from branched necic acids. As established in previous studies, only PAs possessing a 1,2-unsaturated double bond exhibit marked hepatotoxicity and genotoxicity, whereas their saturated analogues are largely non-toxic [19]. This crucial distinction stems from differences in metabolic activation: 1,2-unsaturated PAs undergo bioactivation by hepatic cytochrome P450 enzymes (primarily CYP3A4 and CYP2B6) to generate highly reactive dehydropyrrolizidines (dehydro-PAs). These reactive intermediates then covalently bind to cellular DNA and proteins, which is understood to lead to hepatocyte necrosis, apoptosis, and sinusoidal endothelial injury, ultimately culminating in HSOS [4].

Both the roots and aerial parts of *G. segetum* contain hepatotoxic PAs at concentrations sufficient to cause liver injury, underscoring that the entire plant should not be consumed as medicine or food [20]. The major hepatotoxic PAs identified in *G. segetum* are reported to include senecionine, seneciphylline, and integerrimine. Studies have demonstrated their strong binding affinity to hepatic tissue (e.g., 39.22 ± 1.90 nmol/g liver in rats), providing direct evidence that links PA-induced hepatotoxicity to the formation of pyrrole-protein adducts [21].

**Figure 2:**
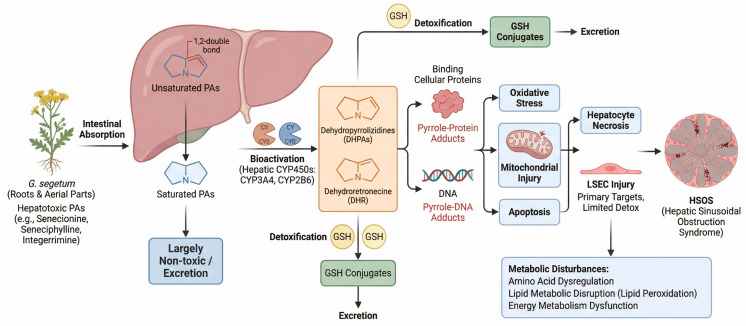
Metabolism of PAs.

Following intestinal absorption, PAs are transported to the liver, where they undergo metabolic activation by cytochrome P450 isoenzymes (primarily CYP2B and CYP3A) to form dehydropyrrolizidine alkaloids (DHPAs). DHPAs are subsequently hydrolyzed to dehydroretronecine (DHR), with both metabolites being highly reactive. These reactive metabolites can conjugate with intracellular glutathione (GSH) to form GSH conjugates, thereby facilitating detoxification of these reactive intermediates. However, the formation of stable pyrrole-protein and pyrrole-DNA adducts by DHPAs and DHR is considered a key molecular event underlying PA-induced hepatotoxicity. These adducts are known to disrupt normal cellular function and initiate oxidative stress, mitochondrial injury, and apoptosis [14,22,23,24]. The rate of pyrrole-protein adduct formation serves as a crucial indicator of PA bioactivation. Furthermore, studies have shown that structural diversity among PAs leads to variable toxicity, with diester-type PAs (e.g., lasiocarpine) typically producing the greatest quantity of adducts and exhibiting the highest hepatotoxic potential [25,26,27]. Exposure to PAs is known to induce widespread metabolic disturbances that exhibit time- and dose-dependent patterns, primarily affecting amino acid, lipid, and energy metabolism pathways. Specifically, amino acid dysregulation impairs hepatic nitrogen handling. Lipid metabolic disruption results in alterations in arachidonic and linoleic acid pathways, as well as elevations in glycerol and triglycerides, contributing to lipid peroxidation and hepatic injury. Moreover, energy metabolism dysfunction, evidenced by abnormal levels of citric acid and lactate, indicates mitochondrial impairment [28]. Liver sinusoidal endothelial cells (LSECs), which are known to express high levels of CYP450 enzymes but possess limited detoxification capacity, are recognized as the primary targets of PA toxicity. LSEC injury compromises the sinusoidal barrier and causes luminal obstruction, which subsequently produces the characteristic pathology of HSOS [29,30,31]. Specifically, Chen et al. demonstrated that following repeated administration of *G. segetum* extract, PA clearance from liver tissue was markedly slower than from serum, with residues detectable up to eight weeks after the final dose. This observed persistence explains the cumulative toxicity of PAs and the continued progression of disease even after discontinuation, thereby underscoring the importance of long-term clinical follow-up in affected patients [32]. Figure 2 illustrates the specific process of hepatic toxin absorption, subsequent liver damage, and detoxification.

## 4. Pathogenesis: From Molecular Events to Pathological Outcomes

### 4.1. Establishment of Experimental Models

**Figure 3:**
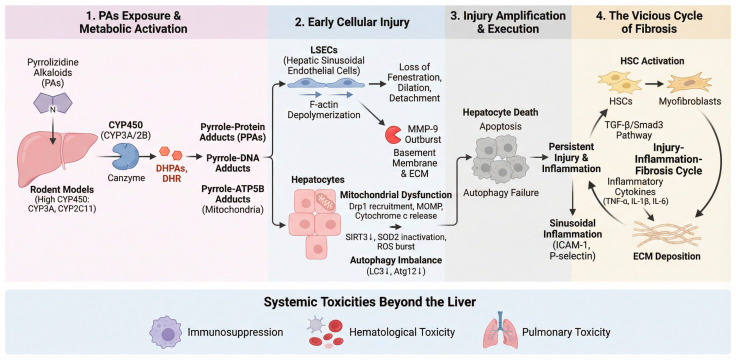
Pathogenesis of PAs-induced HSOS and liver fibrosis.

In vitro studies using hepatic S9 fractions have demonstrated a shorter biological half-life of PAs in rats compared to humans. In human liver microsomes, PAs are primarily detoxified via UGT1A4-catalyzed N-glucuronidation reactions. By contrast, murine and rat microsomal systems lack this specific detoxification pathway. Consequently, CYP450-mediated bioactivation of PAs occurs at significantly higher rates in rodents than in humans, which amplifies their susceptibility to PA-induced hepatotoxicity [33,34]. Rodent models of PA-induced HSOS exhibit pathological features closely resembling those observed in human HSOS. Due to their higher metabolic activation rate and comparable histopathological manifestations, mice have become the preferred species for PA-HSOS model establishment. Male rodents are particularly suited for PA-induced HSOS models, as sex hormones drive high hepatic expression of CYP3A and CYP2C11 enzymes. The synergistic activity of these enzymes markedly enhances PA metabolic activation, enabling the generation of sufficient reactive metabolites even at relatively low exposure doses. This process reproducibly induces characteristic HSOS pathology. The establishment of this model, therefore, provides a robust experimental platform for elucidating the molecular mechanisms underlying HSOS and for identifying early diagnostic biomarkers, such as circulating microRNAs (miRNAs) [35,36]. Figure 3 visually illustrates the progression from hepatic toxin absorption and the formation of toxic metabolites to early liver damage, irreversible hepatocyte necrosis, and fibrosis.

### 4.2. Initiation of Toxic Metabolism and Core Molecular Events

N-oxide metabolites of PAs can be enzymatically reduced back to their parent compounds in both the intestine and liver. Following intestinal absorption, PAs are transported to the liver, where their metabolic activation is predominantly catalyzed by cytochrome P450 enzymes (mainly CYP3A and CYP2B), generating reactive dehydropyrrolizidine alkaloids (DHPAs). DHPAs are subsequently hydrolyzed to dehydroretronecine (DHR) [16]. These electrophilic metabolites form stable pyrrole-protein adducts (PPAs) and pyrrole-DNA adducts with cellular macromolecules. This adduct formation is recognized as the key molecular initiation step of PA-induced hepatotoxicity [3]. Lu et al. further demonstrated that PA metabolites form pyrrole-ATP5B adducts with the β-subunit of mitochondrial ATP synthase. This directly impairs ATP production and precipitates cellular energy failure [14].

### 4.3. Selective Injury to Hepatic Sinusoidal Endothelial Cells

Liver sinusoidal endothelial cells (LSECs) are recognized as the primary cellular targets of PA toxicity. PAs disrupt the cytoskeletal architecture of LSECs by inducing F-actin depolymerization, which leads to dilation, fusion, and eventual loss of fenestrations. Such structural alterations compromise sinusoidal hemodynamics and microcirculatory integrity, as reported by [37]. This damage is highly cell-specific, attributable to the high expression of CYP450 enzymes and relatively limited detoxification capacity within LSECs [38].

### 4.4. Amplification and Execution of Injury: MMP-9 Outburst and Hepatocyte Death

Initial LSEC injury triggers excessive expression and activation of matrix metalloproteinase-9 (MMP-9). This enzyme degrades the endothelial basement membrane and extracellular matrix, thereby exacerbating endothelial detachment and structural disintegration of hepatic sinusoids. As demonstrated in rat HSOS models, administration of MMP inhibitors (MMPi) markedly attenuates sinusoidal damage and intrahepatic hemorrhage [38]. This evidence confirms the pivotal role of MMP-9 in disease progression and highlights its potential as a therapeutic target.

### 4.5. Hepatocyte Death: Mitochondrial Apoptosis and Autophagy Imbalance

Hepatocellular death induced by PA exposure is known to involve both mitochondrial apoptosis and disrupted autophagic regulation. PA exposure promotes the recruitment of dynamin-related protein 1 (Drp1) to mitochondria, leading to excessive mitochondrial fission and fragmentation. This process, as previously demonstrated [39,40] induces mitochondrial outer membrane permeabilization (MOMP), which subsequently leads to cytochrome C release, caspase cascade activation, and the execution of apoptosis. Concurrently, studies have indicated that PAs downregulate the deacetylase SIRT3, causing acetylation-dependent inactivation of the antioxidant enzyme SOD2. The ensuing burst of mitochondrial reactive oxygen species (ROS) further amplifies apoptotic signaling, as reported by Wang, W et al. [41]. While normally cytoprotective, autophagy is markedly suppressed during PA exposure, as evidenced by reduced expression of its key markers LC3 and Atg12. This impaired autophagic flux further aggravates mitochondrial dysfunction and accelerates apoptotic progression [42].

### 4.6. The Vicious Cycle of Fibrosis

Persistent hepatic injury is understood to initiate pathological repair responses characterized by the activation of hepatic stellate cells (HSCs) and excessive deposition of extracellular matrix (ECM) components [43]. The canonical TGF-β/Smad3 signaling pathway is typically activated, driving transcription of profibrotic genes and acting synergistically with inflammatory cytokines such as TNF-α, IL-1β, and IL-6. Notably, PA-induced suppression of autophagy has been observed to coincide with sinusoidal endothelial inflammation, marked by upregulated expression of ICAM-1 and P-selectin. Furthermore, downregulation of the RNA-binding protein CPEB4 has been proposed to represent a novel mechanistic link between PA exposure and autophagy inhibition, thereby reinforcing a self-perpetuating “injury-inflammation-fibrosis” cycle [30].

### 4.7. Systemic Toxicities Beyond the Liver in PAs-HSOS

#### 4.7.1. Immunosuppression

*Gynura segetum* (GS) has been shown to exert marked suppressive effects on both innate and adaptive immunity. Specifically, its effects include the inhibition of macrophage phagocytosis, lymphocyte proliferation, cytokine release, and nitric oxide production—all key components of immune defense. This broad immunosuppressive activity, as reported by Fang, J et al. [44], may predispose affected individuals to heightened susceptibility to infections.

#### 4.7.2. Hematological Toxicity

Exposure to GS has been observed to lead to a generalized elevation in leukocytes (including neutrophils, lymphocytes, monocytes, and eosinophils), concurrently with a marked reduction in platelet count and platelet hematocrit. Furthermore, coagulation assays typically reveal prolonged prothrombin time (PT), activated partial thromboplastin time (APTT), and thrombin time (TT), alongside decreased fibrinogen (FIB) concentration and impaired platelet aggregation. Concomitantly, significant elevations in serum endothelin and nitric oxide levels have been reported. As reported by Song, Z et al. [45], histological analysis of the spleen demonstrates architectural abnormalities, including a reduction of splenic lobules and loss of germinal centers.

#### 4.7.3. Pulmonary Toxicity

Beyond hepatic injury, PAs have also been implicated in pulmonary toxicity. Mechanistic studies indicate that while metabolic activation of PAs is minimal within lung tissue, reactive dehydro-PA metabolites generated in the liver may translocate to the lungs. There, they are believed to form pyrrole-protein adducts that mediate tissue injury. Furthermore, experimental evidence demonstrates that multiple PAs, including those found in *G. segetum*, induce pulmonary lesions in rats at hepatotoxic doses [46,47,48]. This observation suggests that lung injury may represent a shared toxicological feature of PAs.

## 5. Evolution of the Diagnostic Framework: From Clinical Criteria to Precision Biomarkers

### 5.1. Establishment and Optimization of Clinical Diagnostic Standards

The “Nanjing Criteria”, proposed by Chinese experts, provide a pivotal foundation for diagnosing PA-HSOS. These established criteria emphasize four key elements: (1) a documented history of PA exposure; (2) characteristic clinical manifestations including abdominal distension, right upper quadrant pain, hepatomegaly, and ascites; (3) abnormal liver function tests; and (4) typical radiologic findings. As outlined in [49], a comparison with the revised Seattle and Baltimore criteria reveals that the Nanjing criteria incorporate PA exposure history and radiological features, while eliminating requirements for weight gain and specific bilirubin thresholds. This approach enhances their alignment with the clinical manifestations of PA-HSOS. The Roussel Uclaf Causality Assessment Method (RUCAM) is a widely recognized structured framework for evaluating the causal relationship in suspected cases of HILI. Considered the gold standard for causality assessment worldwide, RUCAM enables objective scoring to achieve gradings of probable or highly probable. This method rigorously excludes alternative causes, such as viral hepatitis, autoimmune liver diseases, or other confounders. However, a critical limitation arises if RUCAM is not utilized, as this may result in misdiagnosis where liver injury is erroneously linked to PAs rather than other causes. Conversely, studies suggest that integrating RUCAM with specific biomarkers and criteria, such as the Nanjing Criteria, enhances diagnostic accuracy and is strongly advocated for future validations and clinical practice [50]. A recent diagnostic and prognostic advance is the development of the Drum Tower Severity Score (DTSS). The DTSS system assigns points based on four established parameters: aspartate aminotransferase, total bilirubin, fibrinogen, and portal vein peak velocity. Each parameter is scored according to its value, resulting in a total score ranging from 4 to 16. Patients categorized with mild severity (4–6 points) show a good response to anticoagulation therapy, reportedly exhibiting a negative predictive value as high as 88%. For these patients, outpatient anticoagulation and biweekly follow-up are recommended within the DTSS framework. For those with moderate severity (7–10 points), approximately 45.6% of cases demonstrate no response to anticoagulation; thus, hospitalization and close monitoring are advised, with transjugular intrahepatic portosystemic shunt (TIPS) intervention considered if the initial therapy proves ineffective. In severe cases (11–16 points), the probability of non-response to anticoagulation is 78.3%, leading to a recommendation for direct TIPS to prevent clinical deterioration. As developed by Wang, X et al. [51] the DTSS system accurately predicts the non-response rate to the foundational regimen of supportive care plus anticoagulation. It enables early stratification of disease severity and provides an objective, quantitative tool for guiding clinical decisions—such as identifying high-risk patients who may require early escalation to TIPS—thereby facilitating more precise management of PA-HSOS. Other diagnostic criteria for HSOS are summarized in Table 1.

### 5.2. Advances in Quantitative and Functional Imaging Diagnosis

Imaging plays an indispensable role in the diagnosis, severity assessment, and prognostic evaluation of PA-HSOS. With advancements in computed tomography (CT) and magnetic resonance imaging (MRI), particularly the integration of quantitative imaging analysis, diagnostic radiology has evolved from traditional morphological observation toward functional and precision-based quantification. This paradigm shift provides objective and reproducible diagnostic support that complements and extends the capabilities of conventional clinical tools, such as the Roussel Uclaf Causality Assessment Method (RUCAM) scale and the early “Nanjing Criteria.” Contrast-enhanced CT has emerged as a pivotal modality for identifying and grading PA-HSOS. A large-scale retrospective study by Kan, X. et al. [52] involving 71 patients with PA-HSOS and 222 controls systematically characterized its hallmark CT features: heterogeneous low attenuation of the liver parenchyma (100%), patchy enhancement (92.96%), and hepatic vein narrowing (87.32%). These objective imaging markers, identified in the aforementioned study, strengthen the imaging component of the Nanjing diagnostic framework, enhancing both consistency and clinical applicability. The same study further demonstrated that contrast-enhanced CT outperforms the traditional Seattle criteria in overall diagnostic accuracy.

Quantitative CT analysis enables an objective assessment of disease burden and severity. Notably, Wang, C et al. [53] innovatively applied a threshold-based region-growing algorithm to segment PA-HSOS lesions and calculate the lesion-to-liver volume ratio. Their findings indicated that this ratio showed a strong positive correlation with serum ALT, AST, total bilirubin levels, and clinical severity scores. As a quantitative imaging biomarker, this ratio provides a reproducible and intuitive metric for evaluating disease burden, as described by Wang, C et al. [53] It also compensates for the non-quantitative limitations of RUCAM and the Nanjing Criteria in grading disease severity, thereby offering a more data-driven foundation for treatment planning and prognostic prediction.

The functional role of MRI-specific hepatobiliary contrast agents provides unique insights that enhance both diagnostic precision and prognostic evaluation in PA-HSOS. Specifically, Guo, T et al. [54] demonstrated the distinct value of gadolinium ethoxybenzyl diethylenetriamine pentaacetic acid (Gd-EOB-DTPA)-enhanced MRI for this condition. In their study, all patients exhibited heterogeneous hypointensity of the hepatic parenchyma during the hepatobiliary phase (HBP). This finding reflected impaired hepatocellular function and offered functional imaging evidence for diagnosis. Notably, Guo et al. also observed that the severity of HBP hypointensity correlated positively with prothrombin time (PT) and the international normalized ratio (INR). Furthermore, it was identified as an independent predictor of mortality.

This finding represents a pivotal transition—positioning imaging not merely as a diagnostic modality, but as a quantitative and functional tool for prognostic prediction, extending beyond the capabilities of RUCAM and the exposure-based Nanjing Criteria.

This collective evidence positions imaging not merely as a diagnostic modality but as a quantitative and functional tool for prognostic prediction, extending capabilities beyond those of RUCAM and the exposure-based Nanjing Criteria. Collectively, quantitative CT and functional MRI have ushered PA-HSOS diagnosis into a new era of precision and quantification. Future investigations should therefore focus on: (1) integrating objective imaging parameters (e.g., lesion-to-liver volume ratio, HBP signal intensity) into diagnostic and grading systems to compensate for the non-quantitative limitations of RUCAM and existing standards; (2) exploring the potential of radiomics to uncover latent pathological patterns and prognostic signatures; and (3) developing multi-modal diagnostic frameworks that combine imaging-based metrics with emerging biomarkers such as pyrrole-protein adducts (PPAs) and circulating microRNAs. Such integration may yield a more comprehensive and predictive diagnostic ecosystem for PA-HSOS.

### 5.3. Emerging Frontiers in Precision Biomarkers

#### 5.3.1. Etiology-Specific Biomarker: PPAs

Lin et al. [4] first detected pyrrole-protein adducts (PPAs) in the serum of patients with PA-HSOS. Subsequent work by Ma J et al. [24] demonstrated a strong positive correlation between serum alanine aminotransferase (ALT) levels and hepatic PPA content. This finding established PPA as a shared pathogenic event and a specific biomarker of PA-induced hepatotoxicity across various PA structures. Consequently, quantification of PPAs in serum using ultra-performance liquid chromatography-mass spectrometry (UPLC-MS) has been recommended as an optimal diagnostic biomarker for PA-HSOS [55]. The slow hepatic clearance and persistence of PPAs, as previously reported [34], further explain the cumulative nature of PA toxicity.

#### 5.3.2. Early Diagnostic and Prognostic Biomarkers: microRNAs and Metabolomics

Recent studies have identified significant upregulation of miR-148a-3p, miR-362-5p, and miR-194-5p in both PA-HSOS patients and experimental rat models. Their expression levels correlated positively with the severity of liver injury, indicating strong potential as early, non-invasive diagnostic biomarkers, as reported by Wang, X et al. [37]. Metabolomic profiling has revealed that *Gynura segetum* (GS) exposure induces broad disturbances in amino acid, lipid, and energy metabolism. Specific metabolites, including arginine, creatine, valine, and citric acid, were highlighted as potential biomarker clusters in these studies [30,56]. Furthermore, metabolomic analysis enables clear discrimination between non-toxic *Panax notoginseng* and hepatotoxic *G. segetum*. Ultra-performance liquid chromatography-quadrupole time-of-flight mass spectrometry (UPLC-Q/TOF-MS) analysis of urine and plasma samples revealed distinct endogenous metabolite patterns: L-glutamic acid, L-methionine, cytosine, and L-tyrosine predominated in the Panax group, whereas phytosphingosine, creatine, and sphinganine were enriched in the *Gynura* group. In plasma, key discriminative metabolites identified by Gu, X et al. [30] included arachidonic acid, L-tyrosine, linoleic acid, α-linolenoyl ethanolamide, and lysophosphatidylcholine (15:0) for Panax, versus L-arginine, L-valine, arachidonic acid, and lysophosphatidylcholine [18:2 (9Z, 12Z)] for *Gynura*.

#### 5.3.3. Systems Biology Perspective: The Gut-Liver Axis

A systems-level investigation has revealed a critical link between *Gynura segetum* (GS)-induced hepatotoxicity and gut microbiota dysbiosis. Specifically, GS extracts markedly reduced the abundance of *Lactobacillus* species, which are recognized for their hepatoprotective roles. This perturbed gut microbial composition exhibited close metabolic interactions with host peripheral metabolites, particularly those involved in energy, lipid, and amino acid metabolism. These findings, as discussed previously [30], suggest that GS-induced hepatotoxicity represents a systemic pathological process mediated through the “gut microbiota-host metabolism axis,” rather than a liver-confined toxic response.

## 6. Evolution of Therapeutic Strategies: From Supportive Care to Multimodal Precision Intervention

The “Nanjing Clinical Guidelines” delineate a cornerstone for PA-HSOS management, emphasizing early recognition, timely intervention, and stratified treatment. According to these guidelines, primary principles include: (1) immediate discontinuation of any suspected causative agent to halt ongoing hepatotoxicity; and (2) comprehensive supportive therapy. This therapy aims at symptomatic relief and complication prevention, encompassing hepatic function stabilization, ascites control, coagulation improvement, and infection prevention. For patients with rapidly progressive disease or poor response to standard management, the guidelines further recommend targeted pharmacologic interventions (e.g., anticoagulants and hemodynamic modulators). When indicated, interventional or surgical approaches, such as transjugular intrahepatic portosystemic shunt (TIPS) or liver transplantation, are also advised [49]. This hierarchical and integrative therapeutic framework represents a paradigm shift from empirical supportive care towards evidence-based, precision-oriented, multimodal intervention, marking a new stage in the systematic management of PA-HSOS.

### 6.1. Management of PA-HSOS

With accumulated clinical experience in PA-HSOS, research focus has progressively shifted from establishing diagnostic criteria to optimizing therapeutic strategies, refining prognostic assessment, and advancing the management of special patient populations, encompassing basic supportive therapy and targeted pharmacological interventions.

In the context of autophagy regulation, bicyclol has been shown to exert hepatoprotective effects primarily through restoring autophagic function. Studies in PA-exposed mice have demonstrated that pretreatment with bicyclol significantly reversed the decline in the LC3-II/LC3-I ratio and modulated the expression of the autophagy-suppressive protein Bcl-2, thereby indicating alleviation of PA-induced autophagy inhibition. According to a previous study by Yao, J et al. [44], this protection is mechanism-specific, primarily occurring via the autophagic pathway rather than through apoptosis suppression. Moreover, the study also suggests that bicyclol bidirectionally regulates cytochrome P450 isoenzymes, thereby mitigating the metabolic activation of PAs and indirectly protecting hepatocytes—including sinusoidal endothelial cells—from autophagic dysregulation.

Prednisone is known to act through multiple molecular targets to inhibit inflammation and fibrosis. Its mechanism involves suppressing the activation of inflammatory mediators, such as TNF-α and the transcription factor NF-κB p65, while simultaneously downregulating profibrotic factors including TGF-β1 and CTGF. Consequently, prednisone confers protection during both acute inflammatory and chronic fibrotic stages. As a pivotal transcriptional regulator of inflammation, cell adhesion, and survival, NF-κB is recognized to play a central role in sinusoidal endothelial injury and inflammatory amplification in PA-HSOS [57].

*Salvia miltiorrhiza* (Danshen) has been shown to markedly improve hepatic function and histopathological injury in *G. segetum*-induced HSOS in a dose-dependent manner. Mechanistically, studies have indicated that Danshen treatment significantly reduces hepatic protein levels of TNF-α, VCAM-1, and ICAM-1, and suppresses NF-κB p65 expression. This suggests that its protective effects are mediated through inhibition of NF-κB signaling activation [31].

Ligustrazine has been reported to exert its therapeutic effects by suppressing the expression of early growth response factor-1 (Egr-1) and NF-κB p65, thereby downregulating their common downstream target, tissue factor (TF). This Egr-1/NF-κB/TF signaling axis, identified by Chen, Z et al. [58] suggests that ligustrazine alleviates the procoagulant state and microthrombus formation within hepatic sinusoids, offering a novel molecular target for the management of PA-HSOS-related microcirculatory dysfunction. Table 2 summarizes the treatment guidelines outlined in the Nanjing standard.

### 6.2. Precision Application of Interventional and Surgical Therapies and Advances in Prognostic Evaluation

#### 6.2.1. Anticoagulation Therapy: From Exploratory Use to Established Role and Risk Management

While early management of PA-HSOS primarily relied on symptomatic and supportive care, recent studies have clearly demonstrated the pivotal role of early anticoagulation therapy in promoting disease resolution. A retrospective cohort study involving 49 patients reported a significantly higher remission rate in the anticoagulation group compared to the standard care group (*p* = 0.037) [59]. This conclusion was further validated by an independent study involving 75 patients [60] which demonstrated a significantly higher cure rate (65.3%) in the anticoagulation group compared to the non-anticoagulation group. Consequently, recent advances in clinical understanding emphasize the importance of precise patient selection and dynamic risk balancing between therapeutic benefit and hemorrhagic complications. While anticoagulation significantly improves remission and cure rates, it is recognized to be associated with a bleeding incidence of up to 12.2%. As a result, current clinical practice, informed by these findings [61,62], has shifted away from indiscriminate use toward early, risk-stratified anticoagulation in patients without high bleeding risk. This approach is accompanied by vigilant monitoring and individualized dose adjustment.

#### 6.2.2. Interventional Therapy: Earlier Application and Development of Prognostic Models

For patients with refractory ascites unresponsive to medical therapy, transjugular intrahepatic portosystemic shunt (TIPS) is recognized to have evolved from a last-resort “salvage” intervention to an essential bridging therapy. Multiple studies have demonstrated its remarkable clinical efficacy, showing that TIPS effectively controls ascites, reduces portal pressure, and significantly improves survival outcomes. For instance, one study reported that patients undergoing TIPS exhibited a more than ninefold higher probability of six-month survival compared with those not receiving the procedure [63]. Recent investigations have consequently focused on developing prognostic models to refine postoperative assessment. Specifically, baseline prolongation of prothrombin time (PT) and a serum total bilirubin level exceeding 9 mg/dL on day five after TIPS were identified as independent predictors of mortality by Xiao J et al. [63]. These identified quantitative parameters assist in identifying optimal candidates for TIPS and inform postoperative surveillance strategies, thereby advancing the precision management of interventional therapy.

#### 6.2.3. Combined Procedures and the Expanding Role of Liver Transplantation

In complex cases, direct intrahepatic portocaval shunt (DIPS) combined with inferior vena cava stent placement has demonstrated favorable long-term outcomes, reporting one-, three-, and five-year survival rates of 98%, 89.59%, and 80%, respectively. This combined approach, therefore, offers a viable therapeutic option for patients with outflow obstruction. Meanwhile, liver transplantation is recognized as the definitive treatment for end-stage disease, with its efficacy and indications extensively supported by multiple studies [64].

Notably, emerging research has highlighted patients with underlying liver diseases, such as alcoholic cirrhosis, as a particularly vulnerable subgroup for PA-HSOS. Case reports indicate that PA-HSOS in these individuals is frequently overlooked or misdiagnosed due to overlapping clinical manifestations. Consequently, studies emphasize that in patients with preexisting hepatic disorders, unexplained acute or subacute liver injury or worsening ascites should prompt strong suspicion of PA-HSOS. Targeted diagnostic evaluation, such as liver biopsy, is thus reported to facilitate early identification and intervention, thereby improving prognosis [65,66].

## 7. Summary and Perspectives

Hepatic sinusoidal obstruction syndrome (HSOS) induced by pyrrolizidine alkaloids (PAs) from *Gynura segetum* is the predominant cause of herb-induced liver injury (HILI) in China and parts of Asia. This review systematically synthesizes the molecular pathogenesis, evolution of diagnostic strategies, and therapeutic advances related to PA-HSOS.

Since the introduction of the “Nanjing Criteria” in 2017, clinical research has significantly matured, leading to a paradigm shift in diagnostic approaches towards precision and quantification. Key developments, as comprehensively outlined in this review, include the establishment of the Drum Tower Severity Scoring System (DTSS), advancements in quantitative imaging (e.g., CT lesion-to-liver volume ratio; hepatobiliary-phase signal intensity on Gd-EOB-DTPA-enhanced MRI), and the identification of emerging biomarkers (e.g., circulating microRNAs, pyrrole-protein adducts, metabolomic profiles). These innovations are pivotal for overcoming historical diagnostic ambiguities and enabling earlier, more accurate identification of PA-HSOS.

The Roussel Uclaf Causality Assessment Method (RUCAM), updated in 2016, provides a critical, objective, and quantitative scoring system for assessing causality. While highly valuable, its historical underutilization in early PA-HSOS reports often led to an over-attribution of liver injury to PAs without adequately excluding alternative etiologies (e.g., viral hepatitis, autoimmune diseases, Budd-Chiari syndrome), resulting in misdiagnosis and risk overestimation. Furthermore, plant misidentification (e.g., confusing toxic *G. segetum* with non-toxic Sedum aizoon) further compromised the quality of early diagnostic data. Consequently, integrating RUCAM with specific biomarkers and updated criteria is strongly advocated to enhance diagnostic accuracy and prevent misattributions.

Despite these diagnostic advances, the therapeutic field for PA-HSOS currently faces significant challenges due to a lack of high-quality randomized controlled trials (RCTs). Current interventions—including anticoagulation, TIPS, corticosteroids, autophagy modulators (e.g., bicyclol), and traditional Chinese medicines (e.g., *Salvia miltiorrhiza*, ligustrazine)—primarily rely on retrospective cohorts, case series, or preclinical studies, which inherently yield lower levels of evidence. The absence of large-scale, multicenter RCTs to validate specific agents or regimens significantly limits the development of robust evidence-based guidelines.

Addressing these critical limitations, future challenges should focus on the following:

(1) Integrating RUCAM, pyrrole-protein adduct detection, and DTSS into multimodal frameworks for accurate causality and severity assessment.
(2) Conducting prospective, multicenter RCTs to establish optimal anticoagulation timing, TIPS indications, and the efficacy of novel therapies.
(3) Standardizing botanical identification (e.g., DNA barcoding, chemical fingerprinting) to prevent *G. segetum* confusion with other “San-Qi” herbs.
(4) Enhancing public awareness and regulatory enforcement to curb misuse.
(5) Investigating systemic effects (e.g., gut-liver axis, immunosuppression, pulmonary toxicity) for a comprehensive understanding of long-term sequelae and broader patient impact.

Addressing these historical shortcomings and emerging challenges will be crucial to advancing the precision management of *G. segetum*-related PA-HSOS and improving patient outcomes. Ultimately, a concerted and collaborative effort from scientific, clinical, and regulatory bodies is imperative to eradicate this preventable liver injury.

## Figures and Tables

**Table 1:** Other diagnostic criteria for HSOS.

No.	Standard Name	Year of Publication/Proposal	Institution/Country	Applicable Type	Core Diagnostic Points
1	Seattle criteria (Original)	1984	Seattle bone Marrow transplant Group (USA)	Transplant/Chemotherapy-related HSOS	Within 20 days post-transplant, presence of ≥2 criteria: (1) Jaundice (elevated TBil); (2) Hepatomegaly or right upper quadrant pain; (3) Weight gain > 2%.
2	Baltimore criteria	1987	Johns Hopkins University (USA)	Transplant/Chemotherapy-related HSOS	(1) TBil ≥ 2 mg/dL; (2) Hepatomegaly/right upper quadrant pain; (3) Weight gain > 5%; (4) Ascites-Diagnosis requires TBil plus at least one of the other criteria.
3	Modified Seattle criteria	1993	Multicenter revision (USA)	Transplant/Chemotherapy-related HSOS	Still requires ≥2 criteria within 20 days post-transplant, but now includes imaging and fluid balance assessments.
4	EBMT Diagnostic and severity criteria (Adult)	2016	EBMT (European Society for blood and marrow transplantation)	Transplant/Chemotherapy-related HSOS	(1) Jaundice, hepatomegaly, weight gain, ascites; (2) Occurs within 21 days or may have late onset; (3) Incorporates ultrasound, hemodynamic, and organ function parameters; introduces severity grading.

**Table 2:** Nanjing standard for the treatment of PAs-HSOS.

Treatment Category	Specific Measures	Explanation/Action Mechanism	Precautions
Basic and supportive care	Discontinuation of PAs-containing plants and products	Complete cessation and avoidance of re-exposure to plants containing pyrrolizidine alkaloids and related products	Control disease progression from the source
	Salt restriction and diuresis	Restrict sodium intake (<2 g/day); rational use of diuretics (e.g., spironolactone combined with furosemide)	Basic measures for controlling ascites and alleviating symptoms
	Liver-protective therapy	Administration of hepatoprotective drugs such as polyene phosphatidylcholine, silymarin compounds, and glycyrrhizin preparations	Reduce hepatocyte damage
	Albumin supplementation	Infusion of human albumin to increase plasma colloid osmotic pressure	Applicable for patients with hypoalbuminemia; aids in ascites resolution
	Nutritional support	Provide adequate calories and protein to maintain a positive nitrogen balance	Supports overall patient recovery
Specific drug therapy	Low-molecular-weight heparin anticoagulation	Improves hepatic microcirculation and prevents microthrombus formation	Suitable for early-stage patients without bleeding tendency
	Prostaglandin E1	Vasodilation and inhibition of platelet aggregation	May help improve hepatic blood flow
	Corticosteroids	Suppresses early inflammatory response	Efficacy remains controversial, not recommended for routine use; requires careful benefit-risk assessment by experienced physicians
Interventional therapy	Transjugular intrahepatic portosystemic shunt (TIPS)	Establishes an intrahepatic portal vein-hepatic vein shunt to reduce portal pressure and promote ascites absorption	Indicated for refractory ascites unresponsive to medical diuretic therapy; potential complications include hepatic encephalopathy and shunt stenosis
Surgical treatment	Liver transplantation	Replaces the diseased liver and restores liver function	Applicable to end-stage liver disease patients refractory to all medical and interventional therapies; suitable for irreversible damage such as liver failure and severe cirrhosis

## Data Availability

No new data were created or analyzed in this study. Data sharing is not applicable to this article.

## Abbreviations

The following abbreviations are used in this manuscript.

HSOS	Hepatic Sinusoidal Obstruction Syndrome
Pas	Pyrrolizidine Alkaloids
PPAs	Pyrrole-protein adducts
GS	*Gynura segetum*
HILI	Herb-Induced Liver Injury
DILI	Drug-Induced Liver Injury
LSECs	Liver Sinusoidal Endothelial Cells
DTSS	Drum Tower Severity Score
HSCT	Hematopoietic stem cell transplantation
VOD	Veno-occlusive disease
IARC	The International Agency for Research on Cancer
EFSA	The European Food Safety Authority
MHRA	Medicines and Healthcare Products Regulatory Agency
DHPAS	Dehydropyrrolizidine alkaloids
MMP-9	Metalloproteinase-9
MMPi	MMP inhibitors
DRP1	Dynamin-related protein 1
MOMP	Mitochondrial outer membrane permeabilization
ECM	Excessive deposition of extracellular matrix
PT	Prothrombin time
APTT	Activated partial thromboplastin time
TT	Thrombin time
FIB	Fibrinogen
DIPS	Direct intrahepatic portocaval shunt
TIPS	Transjugular intrahepatic portosystemic shunt
Gd-EOBDTPA	Gadolinium ethoxybenzyl diethylenetriamine pentaacetic acid
RUCAM	Roussel Uclaf Causality Assessment Method
